# Livestock Depredation by Leopards and Tigers Near Bardia National Park, Nepal

**DOI:** 10.3390/ani11071896

**Published:** 2021-06-25

**Authors:** Raj Kumar Sijapati, Hari Prasad Sharma, Sandhya Sharma, Janak Raj Subedi, Jerrold L. Belant

**Affiliations:** 1 Central Department of Zoology, Institute of Science and Technology, Tribhuvan University, Kathmandu 44618, Nepal; rajkumarsijapati999@gmail.com (R.K.S.); janak.subedi@cdz.tu.edu.np (J.R.S.); 2 Nepal Zoological Society, Kathmandu 44618, Nepal; sandhyasharma198@gmail.com; 3 Global Wildlife Conservation Center, State University of New York College of Environmental Science and Forestry, Syracuse, New York, NY 13210, USA; jbelant@esf.edu

**Keywords:** carnivore, livestock depredation, leopard, protected area, tiger

## Abstract

**Simple Summary:**

People in rural Nepal are experiencing increased livestock depredations from large carnivores; however, limited information is available on factors influencing livestock depredations. We quantified potential factors influencing livestock depredations by leopards (*Panthera pardus*) and tigers (*P. tigris*) in and near Bardia National Park (BNP), Nepal. Drivers of carnivore depredations of livestock were influenced by carnivore species, animal husbandry practices, season, and deterrent technique. Leopards killed more livestock than tigers, and the likelihood of livestock depredations was not affected by the number of livestock owned or preventative measures used to reduce depredations.

**Abstract:**

Wildlife attacks on livestock near human settlements are increasing due to the proximity of humans to protected areas. These attacks are often severe due to depredations of livestock adversely affecting the livelihoods of people. The nature of carnivore depredations on livestock can differ based on the carnivore species, animal husbandry practices, season, and deterrent technique. We surveyed people living near Bardia National Park (BNP), Nepal, to compare hoofed livestock depredations by leopards (*Panthera pardus*) and tigers (*P. tigris*) near (<1 km) and far (>1 km) from this protected area. Overall, 1476 hoofed livestock were reportedly depredated by leopards, and 209 by tigers, during 2015–2019. The number of hoofed livestock killed by leopards each season was, at least, 86% higher than the number killed by tigers. More livestock were killed at BNP irrespective of carnivore deterrent techniques used. Due to severe effects created by livestock depredations near BNP, we recommend using more efficacious deterrent techniques when practical, in addition to improved livestock husbandry practices such as night penning.

## 1. Introduction

Large carnivore occurrences near settlements often result in increased conflicts with humans, particularly depredations on livestock [1,2,3]. These conflicts can become more frequent and severe [4,5], particularly in areas with reduced availability of wild prey [6,7,8,9]. With conflicts also due to increasing human populations [7,10,11,12,13] and encroachment into the remaining large carnivore habitats, conflicts with more adaptable large carnivores (e.g., leopard *Panthera pardus*) appear more prevalent than conflicts with species that are less adaptable (e.g., tiger *P. tigris;* [14]). As a consequence of these conflicts, large carnivores experience retaliatory killing which can threaten their persistence [6,15,16]. Due to threats to human safety and property, conservation measures to protect large carnivores can be controversial and may lack support from local communities [7].

People in rural Nepal often use traditional livestock practices for their livelihoods with few alternatives. Following government relocation programs before the 1980s, the number of people living near protected areas has increased. Improved habitat protection, bans on hunting, and public awareness programs have resulted in increased populations of large cats in some protected areas of Nepal [17,18]. Consequently, people who live near PAs in Nepal experience conflicts with wildlife [19,20], though characterization of these conflicts is limited [21]. Human conflicts with large carnivores can increase negative attitudes toward wildlife, particularly when conflicts are severe [22]. People in Nepal have used several techniques to reduce large carnivore attacks on livestock such as establishing barriers to prevent movements of animals [4,23], making loud sounds, and displaying flashing lights. To understand the effectiveness of these techniques to guide management, baseline data on the frequency and severity of conflicts in relation to the use of these techniques are also needed. Human conflicts with large carnivores are increasing in Bardia National Park (BNP), Nepal [8], though knowledge of the extent of these conflicts is limited. We provide baseline data on livestock depredations by leopards and tigers near BNP, characterizing the time of year and frequency they occur, and the efficacy of mitigation techniques to reduce depredations.

## 2. Materials and Methods

### 2.1. Study Area

We conducted this study in the Barahtal Rural Municipality (BRM) buffer zone of Bardia National Park, Karnali Province, Nepal (28.7–28.5 N, 81.3–81.5 E). The buffer zone of BNP comprises 507 km^2^, and the average elevation is about 415 m above sea level. The climate is seasonal and is typically defined as spring (March–May), summer (June–August), fall (September–November), and winter (December–February).

About 70% of the park is forested with dominant plant species including sal *Shorea robusta*, saj *Terminalia alata*, khair *Acacia catechu*, simal *Bombax ceiba*, sissoo *Dalbergia sissoo*, and tooni *Toona ciliata*. The area also supports diverse wildlife including Asian elephants *Elephas maximus*, tigers, leopards, and swamp deer *Rucervus duvaucelii* [24]. There are 400 households in the BRM buffer zone [25] which depend on resources from BNP for their livelihood.

### 2.2. Data Collection

We collected data from 2 January to 12 February 2020 using a questionnaire survey with people living in the BNP buffer zone. We used the number of households in the BRM buffer zone of BNP to estimate the number of households to interview using a 95% confidence interval with a 5% margin of error [26]. We estimated the minimum required sample to be 242 households. From this, we randomly choose 300 households for the questionnaire survey. We compiled all 400 households in an Excel spreadsheet and used the rand() command to select 300 households for the survey.

We interviewed only people >18 years old from a household. We did not discriminate based on education level, gender, ethnicity, or religion. We collected demographic data including age, gender, education (educated: people who attended school through at least grade five; non-educated: people who did not go to school or attended school through grade four or less), family size, and occupation. We asked people whether they experienced conflicts with carnivores and to characterize these conflicts (e.g., frequency and timing, number of livestock lost). We asked them the number of hoofed livestock they owned and the number killed by leopards and tigers during 2015–2019. Respondents reported livestock depredations by leopards or tigers based on their experience, evidence (e.g., leopard or tiger tracks) near the kill site, and evidence on the carcass (e.g., hemorrhaging) including differences between species for killing prey (e.g., leopards suffocate prey as evidenced by bite marks, whereas tigers kill livestock by biting the nape or dorsal portion of the neck). The location of each kill site was recorded, and the distance from each site to the BNP boundary was measured using QGIS. Finally, we asked respondents about the number and type of deterrents used to mitigate the risk of conflicts with leopards and tigers. All aspects of this study were approved by the Department of National Parks and Wildlife Conservation, Nepal (permit DNPWC-67/77-105).

### 2.3. Data Analyses

**Figure 1:**
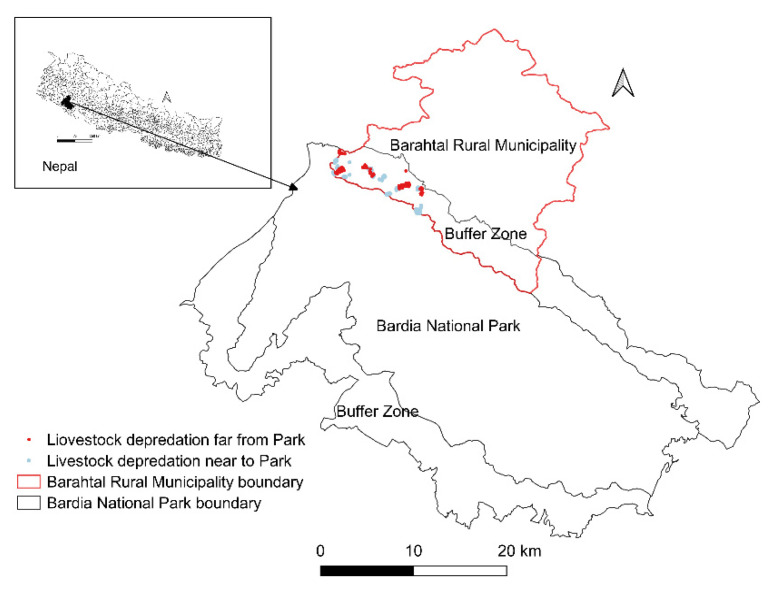
Study area with locations of reported livestock depredations near Bardia National Park, Nepal.

We used chi-square and Kruskal–Wallis tests for binary and numeric responses, respectively, to examine differences between people living near (<1 km from the BNP boundary) and far (>1 km from the BNP boundary) from the protected area. We used the location of each livestock depredation and estimated the distance to the BNP boundary using GIS (Figure 1).

We used generalized linear models with Poisson distribution to identify factors influencing livestock depredation by leopards and tigers using data for 2015–2019. Factors included distance to BNP, number of livestock (cattle and goats) owned, number of techniques used to mitigate conflicts, and season. We defined seasons as spring (March–May), summer (June–August), autumn (September–November), and winter (December–February). We ranked models using the Akaike Information Criterion adjusted for small samples AICc [27], and Akaike model weights to estimate the relative strength of evidence for each model. We considered models with AICc scores within 4 of the most parsimonious models to have support [27]. We conducted model averaging using all models within 4 AICc of the top model to estimate 95% confidence intervals for each variable and accepted statistical significance at α = 0.05. All analyses were performed in the R program [28].

## 3. Results

We interviewed 300 households (147 near BNP and 153 far from BNP); demographic characteristics of respondents near and far from BNP were not different (Table 1). Most respondents were male (66% near BNP and 62% far from BNP) and almost all households (95% near BNP and 95% far from BNP) relied on agriculture for their livelihood. The number of hoofed livestock owned by respondents was also similar between respondents living near and far from BNP. Techniques used to deter leopards and tigers from depredating livestock included shouting, beating pots or drums, shining a flashlight, and firing and ranged from 0 to 4.

**Figure 2:**
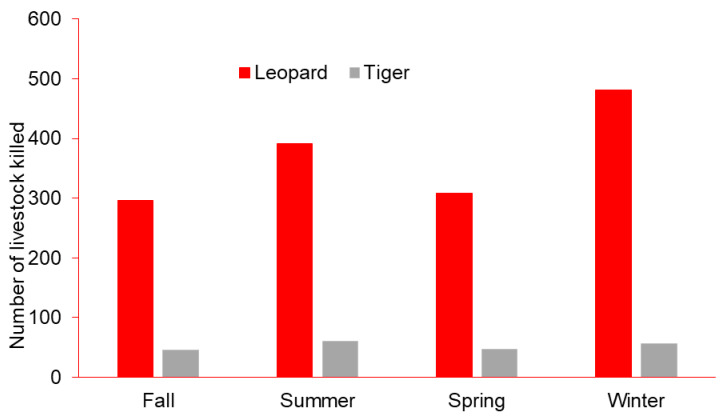
Number of livestock depredations by leopards and tigers seasonally near Bardia National Park, Nepal, 2015–2019.

**Figure 3:**
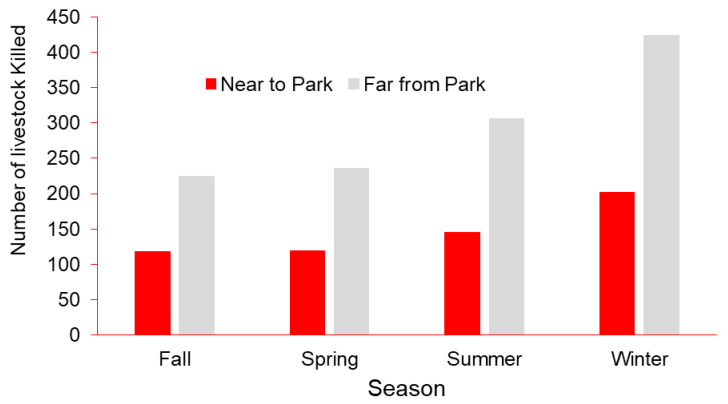
Number of livestock depredations occurring seasonally near (<1 km) and far from Bardia National Park (>1 km), Nepal, 2015–2019.

Overall, 1476 hoofed livestock were reportedly depredated by leopards, and 209 by tigers. Livestock depredation by leopards and tigers was marginally greater far from BNP (Kruskal–Wallis test, *χ*^2^ = 3.681, *p* < 0.055; Table 1). The total number of livestock killed by leopards and tigers differed seasonally (*χ*^2^ = 116.11, df = 3, *p* < 0.001; Figure 2). Most reported depredations occurred in winter (*n* = 626), followed by summer (*n* = 451), spring (*n* = 355), and fall (*n* = 342). More livestock were reportedly killed far from the park boundary during winter (Figure 3).

The best model of livestock depredation events by leopards included distance from BNP and season, and for tigers, it included season; however, all factors assessed were contained in competing models (Table 2). For leopards, livestock depredations were more likely in locations far from the BNP boundary and varied seasonally; the number of livestock owned and the number of preventative measures used to mitigate conflicts did not reduce depredations (Table 3). For tigers, livestock depredations were also more likely to occur far from the BNP boundary, and during seasons other than fall and winter; however, the distance to BNP, number of livestock owned, and number of preventative measures did not influence the probability of livestock depredation.

## 4. Discussion

Leopard depredations of livestock were substantially greater than depredations caused by tigers, irrespective of the season or proximity to BNP. We suspect this marked difference in the frequency of depredations is a consequence of leopards being more common than tigers [29,30]. The fact that more depredations from large carnivores overall occurred near BNP was likely due to the increased number of households moving nearer to BNP since the 1960s [31,32]. Though we found marginally greater numbers of livestock depredations occurring from BNP, the density of livestock depredations (e.g., depredation/km^2^) was greater near BNP, due to the greater abundance of large carnivores [33,34,35]. Large carnivore populations including leopards and tigers have increased in Nepal, especially around PAs [18].

In our study area, more livestock depredations occurred during winter. However, in eastern and western areas of Bardia National Park, more livestock depredations from tigers and leopards were reported during summer and spring [34]. Livestock were kept inside corrals with low walls during winter to shelter them from cold weather (Sijapati, R. K., personal observation). However, within these corrals, animals were tethered which constrained their movements, preventing livestock from avoiding leopards or tigers when confronted by them. This increased vulnerability and crowding of livestock during winter likely facilitated the increased frequency of reported attacks, especially by tigers [36], as identified in other areas of BNP [34]. In addition, wild prey in our study was likely more available to leopards and tigers in summer than winter; wild ungulate mortality by carnivores near BNP was greater during summer [37,38]. Though it is often assumed that carnivores kill ungulates more frequently in winter due to harsh environmental conditions which cause ungulates to congregate [39,40], livestock in our study moved to higher elevations during summer [41] to graze in croplands typically far from BNP where the leopard and tiger abundance is less (Kathayat P., Member Bufferzone User Committee, personal communication). Although livestock are vulnerable to leopards and tigers during summer, we suggest the spatial segregation between livestock and these carnivores limited depredations during summer.

Mitigation techniques used in this study were ineffective in deterring livestock depredations by leopards and tigers. Using lights such as a fox light can be effective as a visual deterrent of leopard depredations of livestock [42]; however, respondents in this study did not use this technique, undoubtedly due to the limited availability of electricity. Additionally, most recorded attacks occurred at night when people were sleeping and unable to monitor livestock or attempt to deter attacks.

Animal husbandry practices can also influence the frequency and occurrence of livestock depredations. Large carnivores, especially leopards, more frequently kill smaller-sized hoofed animals such as goats and calves [36,43]. Goats in our study were typically free-ranging, and attacks on free-ranging livestock by large carnivores are more common [8,44], supporting the high frequency of reported goat depredations by leopards and tigers. Our study area was in the northern portion of BNP where depredations are more common [35]. Greater reported livestock depredations may be related to a reduced prey density from illegal harvest [45], as found previously in BNP [8]. Further, increasing tiger populations in BNP (e.g., tiger abundance increased from 30 to 87 during 1995–2018; [46,47]) may also have contributed to the increased livestock depredations.

Livestock depredations by large carnivores can potentially limit carnivore populations through retaliatory killing [12]. However, we found no evidence of retaliatory killing from local people in the study area that experienced livestock losses (Baduwal, personal communication), possibly, in part, due to people’s belief of the leopard as a cat goddess [2]. Despite livestock losses, most people preferred to live near to BNP because of the greater opportunities for income from tourism, the increased availability of natural resources (both legal and illegal use), environmental services, and esthetic benefits [11,48]. However, the benefits of resource use from BNP may not compensate for the economic losses sustained from livestock depredations. In fact, in our study area, depredations appeared more detrimental to people’s livelihoods because hoofed animals provide their major sources of milk and meat and are crucial for their livelihoods.

## 5. Conclusions

Livestock depredations by leopards and tigers in our study area and other remote areas can be frequent and adversely affect the livelihoods of people. We encourage the use of more effective carnivore deterrent techniques to mitigate this risk. Specifically, we recommend that people in our study area use lighting in and around corrals at night to reduce depredations, particularly in winter when livestock are most vulnerable. Further, we recommend consideration of alternate animal husbandry practices such as keeping mixed livestock (i.e., cattle, buffalo, and goat) in the same corral at night to decrease the vulnerability of smaller-bodied species.

## Figures and Tables

**Table 1:** Attributes of respondents and their livestock living near (<1 km; *n* = 147) and far (>1 km; *n* = 153) from Bardia National Park (BNP), Nepal, 2015–2019. Parameters included age (years), gender (male or female), education (educated: people who attended school through grade five or above; non-educated: people who did not go to school or attended school through grade four or less), occupation (agricultural: if daily life is sustained from agricultural products; or other: if their daily livelihood depends on non-agricultural income), family size (number of individuals), total livestock owned (all hoofed livestock including cattle, buffalo, and goats), and total livestock killed (number of livestock killed including cattle, buffalo, and goats). Range of reported values are in parentheses.

Parameters	Near to BNP	Far from BNP	*p*
Median age	39 (20–88)	43 (20–73)	0.560
Gender (% male)	66	62	0.534
Education (%)	37	28	0.555
Agriculture-basedLivelihood (%)	95	94	0.763
Median family size	7 (3–16)	7 (3–12)	0.855
Median livestock owned	14 (2–38)	14 (2–37)	0.126
Median livestock killed	5 (0–22)	6 (2–16)	<0.055

**Table 2:** Generalized linear models to identify factors related to livestock depredations by leopards and tigers, Bardia National Park, Nepal. Number of livestock killed was used as the response variable; total livestock owned (number); preventive measures (number of techniques used to deter tigers and leopards, 0–4). Season (number of livestock killed: fall, winter, spring, summer), park distance (near: <1 km, and far: >1 km from the park boundary). K is the number of parameters, ∆AICc is the difference between the AICc value of the best supported model and successive models, and w_i_ is the Akaike model weight.

Species	Covariates	K	∆AICc	Wi
Leopard	Distance to park + Season	6	0.00	0.26
	Distance to park + Livestock owned + Season	7	0.19	0.24
	Season	5	0.82	0.17
	Livestock owned + Season	6	1.32	0.14
	Distance to park + Preventive measures + Season	7	1.99	0.1
	Distance to park + Preventive measures + Season + Livestock owned	8	2.17	0.09
	Null	1	334.9	0.00
Tiger	Season	4	0.00	0.23
	Livestock owned + Season	5	0.89	0.15
	Preventive measures + Season	5	1.46	0.11
	Distance to park + Season	5	1.97	0.09
	Preventive measures + Livestock owned + Season	6	2.36	0.07
	Null	1	177.5	0.00

**Table 3:** Model-averaged parameter estimates and lower and upper 95% confidence limits describing livestock depredated by leopards and tigers, Bardia National Park, Nepal. Parameter estimates were averaged from all models reported in Table 2. * Significant effects are in bold.

Species	Covariates	Estimate	SE	Lower Limit	Upper Limit	z	p *
Leopard	(Intercept)	0.888	0.078	0.735	1.042	11.342	**<0.001**
	Fall	0.116	0.025	0.067	0.165	4.64	**<0.001**
	Spring	0.139	0.025	0.091	0.188	5.594	**<0.001**
	Summer	0.102	0.022	0.058	0.146	4.54	**<0.001**
	Winter	0.109	0.017	0.075	0.144	6.243	**<0.001**
	Livestock owned	−0.004	0.003	−0.011	0.002	1.298	0.194
	Preventive measures	0.002	0.021	−0.039	0.044	0.111	0.911
	Distance to park	−0.092	0.053	−0.197	0.013	1.718	0.086
Tiger	(Intercept)	−1.849	0.175	−2.191	−1.507	10.589	**<0.001**
	Distance to park	−0.022	0.145	−0.307	0.263	0.152	0.879
	Fall	0.296	0.062	0.175	0.418	4.792	**<0.001**
	Spring	−0.022	0.0721	−0.165	0.119	0.315	0.752
	Summer	0.227	0.057	0.116	0.338	4.015	**<0.001**
	Winter	0.221	0.043	0.137	0.305	5.155	**<0.001**
	Livestock owned	0.009	0.009	−0.008	0.026	1.05	0.294
	Preventive measures	−0.044	0.061	−0.163	0.074	0.732	0.464

## Data Availability

The data presented in this study are available on request from the corresponding authors.
